# Effects of a Short-Term Slackline Training Program on Energy Expenditure and Balance in Healthy Young Adults: A Preliminary Report of a Randomized Controlled Trial

**DOI:** 10.3390/ijerph19084830

**Published:** 2022-04-15

**Authors:** Sebastian Rutkowski, Adam Wrzeciono, Oliver Czech, Anna Rutkowska, Jan Szczegielniak

**Affiliations:** 1 Department of Physical Education and Physiotherapy, Opole University of Technology, 45-758 Opole, Poland; a.rutkowska@po.edu.pl (A.R.); j.szczegielniak@po.edu.pl (J.S.); 2 Department of Physiotherapy, University School of Physical Education in Wrocław, 51-612 Wrocław, Poland; awp97adam@wp.pl (A.W.); oliverek8@o2.pl (O.C.)

**Keywords:** exercise, physical exertion, postural balance

## Abstract

The development of technology and a fast-paced lifestyle has caused a significant decrease in physical activity, especially among young people. These worrying trends can be countered by the use of attractive forms of physical recreation, including the increasingly popular slackline. The aim of this study was to evaluate energy expenditure during slackline training and to analyze changes in dynamic and static balance parameters after supervised slackline training sessions. The study enrolled 28 healthy volunteers (14 men and 14 women aged 21–25) who were randomly divided into two groups: experimental and passive control. The energy expenditure level was the primary outcome and was assessed using the SenseWear Armband. Each participant underwent an initial and final balance assessment using two selected protocols on the Balance Master platform. The intervention lasted 5 days, with 15 min of supervised training per day. The average energy expenditure expressed in MET was 6.0 (±0.7) MET per training session. An analysis of the results regarding static and dynamic balance showed that the group participating in slackline training significantly improved stability on foam surfaces with their eyes open (*p* < 0.003), as well as tandem walk speeds (*p* < 0.05), both with small effect sizes. The results suggested that slackline training has the potential to produce significant positive effects on general health statuses following the World Health Organization’s (WHO) recommendations on physical activity. The significant improvement in task-specific balance suggests that slackline training could become an important element of the prevention and rehabilitation of many injuries.

## 1. Introduction

A significant decrease in physical activity can be observed as a global trend in the 21st century, especially among young people. It is estimated that one-third of the population does not lead an active lifestyle today, and living a sedentary lifestyle is defined as a disease of the 21st century [1]. The development of technology and a fast-paced lifestyle likely affected this behavior [2]. The World Health Organization’s (WHO) guidelines recommend moderate-intensity physical activity for healthy people between 18 and 64 years old. This involves undertaking moderate-intensity aerobic training 5 days a week for 30 min or undertaking high-intensity activity 5 days a week for 15 min [3]. WHO guidelines report that moderate energy expenditure is in the range of 3–6 metabolic equivalents of task (MET). A level below 3 MET is considered mild physical activity, and high-intensity activity is an expense of above 6 MET. Therefore, the exploration of sources of easily accessible, motivating forms of physical activity seems justified.

Slacklining has become increasingly popular as an exercise-related activity. This recreational activity combines the elements of gymnastics, acrobatics, and mountain climbing. The main aims of the activity are to maintain balance and perform different gymnastic moves while crossing a 2.5–5 cm wide nylon tape hung above the ground. The available materials demonstrate difficulties in starting training, and beginners can normally neither enter the line without assistance nor maintain balance. This discipline can be classified as a fun sporting activity and may be more enjoyable and even more demanding in some aspects than classical balance training [4]. Previous research indicates a positive effect of slackline training on postural stability [5], improving balance [6,7,8], neuromuscular control [9], and concentration or motivation [6]. A meta-analytical review by Donath et al. revealed strong evidence that slackline training induced task-specific balance adaptations with small to moderate transfer effects to static and dynamic standing balance tasks [10]. However, the meta-analysis assessed only the pooled effects of 4–6 weeks of slackline training alone on static and dynamic balance performance; thus, there is a lack of research describing the effectiveness of short-term training.

Postural balance is the ability to keep the body in equilibrium and to regain balance after the shift of body segments [11]. It is believed that many injuries result from dysfunctions in balance ability [12]. In an attempt to decrease the risk of injury, balance improvement exercises are part of different training programs for professional athletes [13]. It is assumed that slackline training could complement standardized neuromuscular training programs [9] and improve task-specific balance [10]. Slackline offers a relatively small base of support, and the tape itself sways in medio-lateral directions [14]. As a result, slackline training can be considered as whole-body proprioception training on an unstable surface. Therefore, proprioception may be improved, which will translate into an overall improvement in postural stability. Thus, it is interesting to consider how slackline training affects dynamic and static balance parameters and whether it could be a useful tool in injury prevention or recovery processes.

An analysis of PubMed and Scopus scientific databases showed no research on energy expenditure during slackline training. It seems interesting to consider whether slackline training can be an adequate physical activity that can provide health benefits by meeting WHO recommendations. Therefore, the main aim of the study was to determine energy expenditure during fifteen-minute training sessions. The second objective was to assess changes in balance parameters after a 5-day training program and to analyze the strengths and weaknesses of this approach in injury prevention or rehabilitation treatment. We hypothesize that the short-term slackline training program would improve participants’ static and dynamic balance parameters.

## 2. Materials and Methods

### 2.1. Design

**Figure 1:**
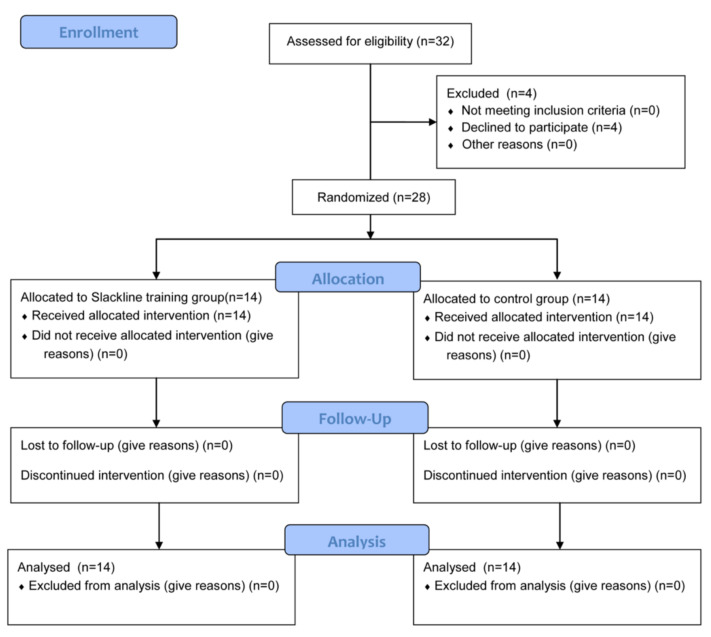
CONSORT flow diagram.

This trial was designed as a parallel-group study. Participants were randomly assigned to two groups, with 14 subjects in the experimental group and 14 subjects in the control group. The randomization (ratio 1:1) sequence was generated at the start of the trial using a computerized block randomization generator (randomizer.org, accessed on 10 June 2019). The allocation sequence was concealed from the principal investigator enrolling participants in sequentially numbered, opaque, sealed envelopes. A single blind randomization was applied to assessors, assuring blindness to treatment allocation and randomization procedures. Before the first training session and 30 min after the last training session, a balance assessment was conducted. Participants in both groups were instructed to not practice any form of balance training during the duration of the study. Each participant provided written consent to participate in the study. The research was conducted in accordance with the Declaration of Helsinki. The study was approved by the Bioethical Commission of the Opole Chamber of Physicians (Resolution No. 289 of 7 June 2019) and was registered in anzctr.org.au (accessed on 11 December 2019) (ACTRN12619001761156). The study was designed in accordance with the recommendations of the CONSORT statement (Figure 1).

### 2.2. Participants

This study enrolled 28 healthy volunteers. Posters encouraging participation in the research were hung at the university. The basic characteristics of the groups are shown in Table 1. The inclusion criteria were males and females aged 19–25 who did not engage in regular physical activity > 5 h a week. Exclusion criteria included diseases and injuries related to the locomotor system that impaired the function of locomotion, surgical procedures, diagnosed chronic disease, and hospitalization within 24 months before the study. None of the participants had prior training experience. Before inclusion in the study, the participants had an initial interview during which they were asked about situations that could exclude participation in the study according to the exclusion criteria. The data collected from human subjects were managed and protected by the research team.

### 2.3. Intervention

**Figure 2:**
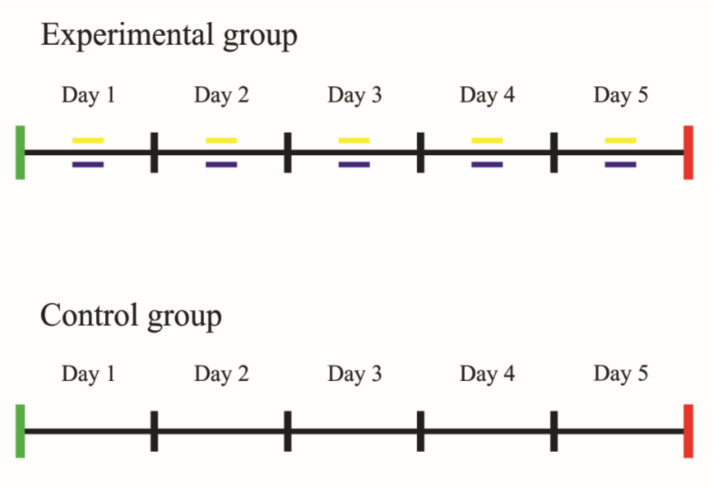
The flow of study interventions. Green (Day 1): eligibility criteria, informed consent, and baseline balance assessments (mCTSIB, Tandem Walk Test). Red (Day 5): end of intervention, follow-up balance assessment (mCTSIB, Tandem Walk Test). Yellow (Day 1–Day 5): slackline training sessions. Blue (Day 1–Day 5): SenseWear Armband activity.

After the initial balance assessment, participants in the experimental group participated in a five-day monitored slackline training. Each session lasted 15 min. The participants’ task was to maintain balance as long as possible and to cover the longest possible distance crossing a 5 cm wide and 9 m long nylon tape stretched at a height of 50 cm. For slackline training, we used Gibbon CLASSIC LINE 15 M tape, the most universal tape offered by Gibbon Slacklines (Gibbon Slacklines GmbH, Stuttgart, Germany). Each step-off caused a return to the beginning of the tape. The procedure was repeated until the training endpoint—for 15 min. The control group participants did not undertake specific tasks after the initial assessment. The passive control group was selected to consider whether slackline training would result in significant changes in balance parameters. During training and testing, the participants were barefoot. During the intervention, all security measures were taken. Training sessions were held indoors in a sports hall. The slackline was stretched between two structural poles secured with a thick sponge. Four-meter-wide and ten-centimeter-thick gymnastic mattresses were placed under the entire length of the nylon rope. There were no dangerous obstacles (e.g., walls and pillars) within 4 m of the intervention point. After the intervention was completed, a final balance assessment was conducted. We noted full adherence by the study’s participants. The participants in both groups took part in the examination, which consisted of the same tests as the initial assessment, i.e., the mCTSIB and the Tandem Walk Test. The flow of the study intervention is presented in Figure 2.

### 2.4. Outcomes

Assessment of energy expenditure during slackline training was the primary outcome. As a secondary outcome, we evaluated participants’ balance.

#### 2.4.1. Energy Expenditure

Energy expenditure was assessed using the SenseWear Armband Pro 3 (BodyMedia, Inc., Pittsburgh, PA, USA). The device allows for the evaluation of energy expenditure, expressed in metabolic equivalent of task (MET) and kilocalories (kcal), and monitoring parameters such as the total number of steps, level and duration of physical activity, body temperature, duration of sleep, and rest period. The device was active every day during training. The intensity of physical activity is divided into four categories: sedentary (<1.5 METs), light intensity (1.5–2.9 METs), moderate intensity (3–6 METs), and intensive intensity (>6 METs) [15]. Several studies have shown the accuracy, usability, validation, and test–retest reliability of the device for estimating energy expenditure during physical and everyday activities [16,17].

#### 2.4.2. Balance

Each participant underwent an initial balance assessment. The examination was conducted using two selected protocols available on a computer posturography system to evaluate dynamic and static balance with NeuroCom Balance Master (NeuroCom International, Inc., Clackamas, OR, USA). The first was mCTSIB (Modified Clinical Test of the Sensory Interaction on Balance), which specifies the postural swing speed measured as degrees per second (°/s) in 4 different conditions. The test allows for the evaluation of how well the examined participant uses sensory inputs when one or more sensory systems are compromised. In the first trial (Firm EO), testing was conducted with the participants on stable ground with open eyes, so vision, somatosensory, and vestibular systems were available to maintain balance. In the second condition (Firm EC), when the participant was standing on a firm surface with closed eyes, only the somatosensory and vestibular systems allowed the maintenance of balance. In the third condition, (Foam EO), the participant was standing on the foam surface with open eyes. In the fourth condition (Foam EC), the participant was standing on the foam surface with closed eyes. The test consists of three trials in each condition with the same course. The test result is the mean of all 3 measurement trials. Each failed attempt was marked as “interrupted” and repeated. The mCTSIB overall demonstrated strong test–retest reliability [18]. The second assessment protocol was the Tandem Walk Test, which identifies the dynamic balance characteristics of a participant crossing a measurement platform using heel-to-toe tandem walking. The measured parameters were the step width measured in centimeters (cm), which was the lateral distance in centimeters between the left and right feet on successive steps; speed measured as centimeters per second (cm/s); and end sway measured as degrees per second (°/s), which was the velocity in degrees per second of the anterior/posterior component of COG sway for 5 s beginning when the patient terminates walking. The test consists of three trials with the same course. The three trials were performed with a 10-s rest between trials. The test result was the mean of all 3 measurement trials. Each failed attempt was marked as “interrupted” and repeated. Tandem gait outcome measures demonstrated high test–retest reliability [19]. Protocols available on the Balance Master were used in studies on the development of postural control in 6–17-year-old healthy children [20] and balance disorders in patients with various disease entities [21,22].

### 2.5. Statistical Analysis

The results were collected in an Excel spreadsheet and subsequently subjected to statistical analysis using STATISTICA 13 software (StatSoft, Cracow, Poland). The sample size was calculated based on the results of the meta-analysis according to the effectiveness slackline training on dynamic balance performance [10] with an effect size of 0.52. We used G*power 3.1.9 software (Universität Düsseldorf, Düsseldorf, Germany) to calculate the sample size. The calculation was based on the F test and repeated measures between factors: The type I error rate was set at 5% (alpha-level 0.05), the effect size of the main outcomes was 0.52, and the type II error rate produced 85% power. It was determined that 28 patients should be enrolled. With respect to the basic descriptive characteristics, the arithmetic mean and standard deviation were derived. As normality tests (Lilliefors test) revealed that none of the balance trials and energy expenditure measurements followed a normal distribution, nonparametric tests were used. The baseline characteristics of the groups were compared using the Mann–Whitney U test. The difference in the mean values for the mCTSIB and the Tandem Walk Test was assessed with the Mann–Whitney U test. The difference in the energy expenditure values between training days was assessed with the Kruskal–Wallis test. The effect sizes were calculated with Cohen’s d. An effect size ≥ 0.20 was considered small, while an effect size ≥ 0.50 was considered medium and an effect size ≥ 0.80 was considered large [23]. The statistical significance of the results was accepted at *p* < 0.05.

## 3. Results

Age, sex, and BMI characteristics were similar for the experimental and control groups (*p* > 0.05). The analyzed data were obtained from 28 participants.

### 3.1. Energy Expenditure

**Figure 3:**
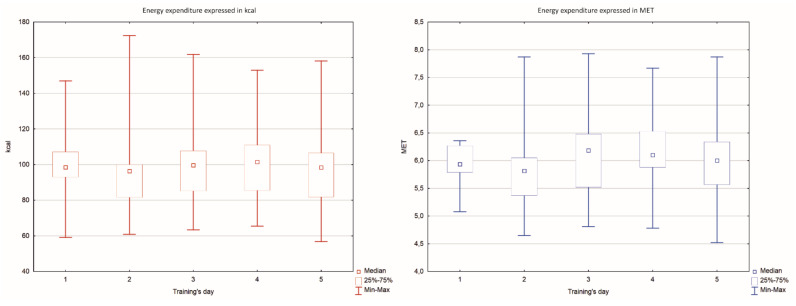
Energy expenditure results. No significant difference between training’s days was found (*p* > 0.05). MET: metabolic equivalent; kcal: kilocalories.

Analysis of the results showed that during 5 days of training, the total average energy expenditure was 552 kcal, approximately 100 (±22.5) kcal per training session. There were no differences between the sessions (*p* < 0.64). The average energy expenditure expressed in MET was 6.0 (±0.7) MET. There were no statistically significant differences in the MET values between the sessions (*p* < 0.53) (Figure 3).

### 3.2. Balance

The analysis of the initial balance assessment showed no statistically significant differences between the study groups on the mCTSIB test and Tandem Walk Test.

The analysis of the data showed significant differences in postural sway velocity (°/s) under sensory conditions on a foam surface with eyes open between experimental groups when analyzing delta values (posttest values minus pretest values) (*p* < 0.003) with a small effect size (d = 0.37). The percentage difference between groups was 333%. The remaining parameters evaluating the static balance revealed no statistically significant changes. The analysis of the dynamic balance data showed significant differences in the tandem walk speed parameter (*p* < 0.050) with a small effect size (Table 2). The percentage difference between groups was 101%.

## 4. Discussion

The design of this study is unique in terms of the duration of the experiment. Slacklining can be practiced almost anywhere, and the preparation of a training environment takes only a few minutes. For most slackline enthusiasts, this activity is more like a hobby than a competitive sport. Therefore, this study focused on determining the short-term benefits of slackline training sessions.

The main aim of the study was to determine energy expenditure during fifteen-minute training sessions. The first hypothesis was that fifteen-minute slackline training would produce a beneficial impact on general health due to intensive energy expenditure, as recommended by WHO. The results showed that the average energy expenditure during slackline training was 6.0 (±0.7) MET. Thus, it seems that the results obtained are on the threshold of an intensive level, although not all subjects exceeded this value every day. The energy expenditure level did not differ significantly between training sessions. According to WHO guidelines, physical activity > 6 MET is defined as high intensity. Therefore, the results of our study suggest that 15-min slackline training, with an appropriate participant commitment, has the potential to meet the WHO recommendations for the duration and high intensity of weekly physical activity for people aged 18–64 years and, thus, affects general health. Such results partially support the first hypothesis.

The second aim was to assess changes in balance parameters after a 5-day training program. We hypothesized that the short-term slackline training program would have a positive effect on the participants’ static and dynamic balance parameters. The results showed significant improvements in balance performance while standing on a foam surface with eyes open measured within the mCTSIB trial. The similarity between the training and assessment conditions seems to explain why static balance on an unstable surface improved. Maintaining the balance on the rope resembles the measurement conditions on unstable grounds in the FOAM EO protocol. This may be caused by training the sensory-motor and vestibular skills during slackline training. Moreover, the effect of task-specific improvement in sway velocity under foam surface conditions may result in improved sway velocity under firm surface conditions due to improvements in sensory-motor skills as a task-specific effect. The next significant improvement was noted in the tandem walk speed. This may also be the result of the similarity between training and measurement conditions. Similar movement patterns are used when crossing the slackline and crossing the measuring platform during the Tandem Walk protocol on the NeuroCom Balance Master. Thus, the second hypothesis was confirmed, and improvements in static and dynamic balance parameters can be called task-specific effects due to similar training and assessment conditions.

The available literature does not indicate research related to energy expenditure during slackline training and different sport activities in young healthy people. Therefore, we compared the results of our research to other different physical activities popular in a similar age group. Monedero et al. evaluated the physical activity levels in young people during different types of active video gaming. An average energy expenditure was estimated as 5.1 MET during entertainment-themed games and 6.4 MET during fitness-themed games [24]. The study proves that active games can be an interesting alternative to traditional physical exercises. Frappier et al. examined energy expenditure in young people during sexual activity and compared it to energy expenditure during a 30-min endurance exercise session. The average energy expenditure of the sexual activity was 101 kcal or 6.0 MET in men and 69 kcal or 5.6 MET in women. The average energy expenditure during the training session was 276 kcal or 8.5 MET in men and 213 kcal or 7.1 MET in women. The research results indicate that sexual activity with an average energy expenditure of 85 kcal or 5.8 MET can potentially be considered as an important exercise [25].

An interesting issue is energy expenditure during simple everyday activities and professional work that is considered hard. Ohkawara et al. investigated the parameters of physical activity during household and different locomotive activities. Tasks such as vacuuming, dishwashing, or washing dishes are considered light-intensity activities (1.5–2.9 MET). However, many daily actions such as walking at different speeds, descending stairs, or moving a small load are considered moderate intensity activities (3–5.9 MET) [26]. On the other hand, Gram et al. investigated occupational physical activity parameters in construction workers. The average level of physical activity intensity during work was estimated to be 1.8 MET [27]. This finding suggests that working professionally and carrying out daily duties is not enough to meet the WHO guidelines for the duration and intensity of weekly physical activity, and additional physical activity is necessary to stay healthy. The results of our study may be of significant practical importance because they suggest that 15-min slackline training could be an attractive and interesting recreational physical activity that has the potential to benefit general health by preventing the development of civilization diseases.

Previous studies on slackline training have reported significant improvements in several postural control variables. Our findings are in line with the results of Donath et al.’s meta-analysis. Slackline training demonstrated meaningful task-specific training effects in balance performance tasks that are closely related to training content [10]. Interesting results were obtained by Mildren et al. After a week-long training session, coordinated movement synergies within the upper body were observed in the subjects, which allowed the participants to maintain and regain balance during demanding slackline training [8]. Such results show the effectiveness of slackline training in building task-specific effects in balance performance. Extending traditional balance training with slackline training could have a beneficial effect on the effectiveness and rate of obtaining results. Volery et al. investigated changes in balance and sensory-motor skills. Fifteen-minute training sessions occurred 3 times a week for 6 weeks. Slackline training has been shown to be partially complementary to conventional sensorimotor training and can be a motivator for more efficient training [7]. Significant improvements in various parameters after slackline training were shown by Fernández-Rio et al. The study investigated the effects of slackline training in young soccer players on acceleration, agility, jump performance, and postural control. The final measurements after a six-week supervised slackline training showed a significant improvement in all assessed parameters [28]. According to Donath et al., daily training using the slackline, supported by traditional balance exercises, can improve static and dynamic balance [5]. The difference in training duration makes it difficult to compare the results. However, even our short-term training seems to have the potential to positively affect task-specific balance.

An additional objective of the project was to analyze the strengths and weaknesses of this approach when considered as an injury prevention or rehabilitation treatment. Many injury prevention training programs are based on balance, sensorimotor, and proprioceptive exercises [18,19]. The positive impact of slackline training on static and dynamic balance as well as complementarity with conventional sensorimotor training could indicate the usefulness of slackline training in injury prevention. Granacher et al. investigated the impact of slackline training in reducing the risk of sports injuries as a result of increasing strength and balance. Based on the results, the authors concluded that slackline training without the support of other exercises was found to have no injury prevention effects [29]. In contrast, the results of our study suggest that short-term slackline training has the potential to improve proprioception, as we noted statistically significant improvements in static balance on unstable surfaces and dynamic balance in tandem walk tests. Therefore, a lower training duration cannot be ruled out as leading to beneficial training effects. However, a higher training dosage would conceivably increase the effects and, moreover, would be needed to benefit everyday life situations [30].

Another important aspect of balance-based and sensorimotor-based training is that it involves widely understood rehabilitation. The quick restoration of disturbed balance and motor functions is important in sports as well as in many fields of medicine (e.g., orthopedics and neurology). Thomas et al.’s study showed that slackline training has a positive impact on postural control in older adults and could be a useful tool in reducing the risk of falls [30]. González et al. indicated that slackline training may improve static postural control and motor skills in adolescents and children suffering from spastic cerebral palsy [31]. Such results indicate the need to investigate the impact of slackline training as a complementary therapy of the rehabilitation process in various neuromotor diseases. During our study, we observed full adherence to the program. All participants were eager to participate in subsequent trainings. We did not record any serious falls (forward or backward) during training. It was natural to step off the tape due to a loss of balance, but these occurrences were calmly accepted by the participants. The subjective evaluation of the participants indicated high satisfaction; however, we did not use objective research tools for this purpose.

### 4.1. Limitations

Although this study provides encouraging results, we recognize that some limitations should be considered. One limitation is that the study did not evaluate the strength of the lower limbs, which may influence the learning of a balance task; this should be taken into account when interpreting the effects of balance training [32]. Second, the short training period may have resulted in a small difference between the groups. Another limitation is the final assessment timepoint. Thirty minutes after training, the adaptation mechanisms could still be working, which could have influenced the results of the balance measurements. Therefore, a follow-up assessment could provide additional valuable information about the benefits of slackline training on dynamic balance. Furthermore, this study enrolled a small sample size. However, a small number of participants were used to initially assess the occurrence of possible changes in the measured parameters and to assess the appropriate design of the intervention. 

### 4.2. Practical Applications

The results suggest that short-term slackline training has the potential to produce high-intensity energy expenditure. As a result, this activity can be considered an attractive and effective supplementary recreational activity, making daily physical activity more varied and attractive. Considering the benefits on human balance, slackline training has the potential to be considered as part of injury prevention programs and rehabilitation processes. Perhaps a good solution is to include slackline training as a complementary method to traditional training and rehabilitation programs.

## 5. Conclusions

The results showed that short-term slackline training has the potential to produce high-intensity energy expenditure, therefore following the WHO recommendations on physical activity. Furthermore, the results of this study confirmed previous reports indicating an improvement in task-specific balance (standing on unstable ground and tandem walk speed). The promising results shown may guide the future startup of randomized controlled studies that can show the efficacy of slackline training. The use of slacklines in injury prevention programs and rehabilitation processes should be considered in future studies. However, it is necessary to extend the training program so that slackline training complements traditional training or rehabilitation.

## Figures and Tables

**Table 1:** Group characteristics.

	Experimental Mean (SD)	Control Mean (SD)
Age (y)	22.4 (1.5)	22.3 (0.8)
Male/female	7/7	7/7
Hight (m)	1.7 (0.1)	1.7 (0.2)
Body weight (kg)	67.0 (14.1)	68.2 (10.3)
BMI (kg/m^2^)	22.8 (3.6)	22.0 (1.8)

Notes: BMI: body mass index; SD: standard deviation.

**Table 2:** Comparison of static and dynamic balance parameters in the experimental and control groups.

Variables	Experimental Group (n = 14)			Control Group (n = 14)			*p*	Effect Size d
	Pre	Post	Δ Post-Pre	Pre	Post	Δ Post-Pre		
Modified clinical test of sensory interaction on balance								
Sway velocity on firm surface, eyes open (°/s)	0.2 [0.1–0.3]	0.2 [0.2–0.3]	0.0	0.2 [0.2–0.3]	0.3 [0.2–0.4]	0.00	0.792	0.32
	0.22 (0.13)	0.24 (0.11)	0.03	0.26 (0.15)	0.31 (0.16)	0.05		
Sway velocity on firm surface, eyes closed (°/s)	0.2 [0.1–0.3]	0.2 [0.1–0.3]	0.0	0.2 [0.2–0.3]	0.2 [0.1–0.3]	0.00	0.658	0.03
	0.23 (0.13)	0.21 (0.12)	−0.02	0.24 (0.12)	0.23 (0.12)	−0.01		
Sway velocity on foam surface, eyes open (°/s)	0.4 [0.3–0.5]	0.3 [0.3–0.4]	−0.1	0.3 [0.4–0.4]	0.4 [0.3–0.5]	0.00	0.003 *	0.37
	0.47 (0.49)	0.34 (0.13)	−0.24	0.35 (0.14)	0.41 (0.18)	0.06		
Sway velocity on foam surface, eyes closed (°/s)	0.4 [0.4–0.6]	0.5 [0.4–0.5]	0.0	0.4 [0.3–0.6]	0.4 [0.3–0.6]	−0.05	0.854	0.10
	0.49 (0.21)	0.46 (0.15)	−0.03	0.45 (0.21)	0.43 (0.19)	−0.02		
Tandem Walk Test								
Step width (cm)	6.55 [6.2–7.60]	6.9 [6.4–7.7]	0.2	6.5 [6.1–7.2]	6.0 [4.8–7.2]	0.4	0.365	0.40
	6.88 (0.98)	6.87 (1.10)	−0.01	6.11 (1.43)	6.62 (1.08)	0.39		
Tandem walk speed (cm/s)	17.7 [13.7–23.8]	20.3 [17.9–24.4]	1.9	20.4 [17.2–24.2]	18.4 [13.9–25]	−1.35	0.050 *	0.30
	18.81 (5.98)	21.27 (4.77)	2.46	19.27 (6.64)	20.94 (4.28)	0.81		
Tandem end sway (°/s)	3.4 [2.7–4.4]	3.3 [2.4–4.3]	−0.2	2.25 [2.0–3.1]	2.6 [1.9–4.0]	−0.35	0.192	0.25
	3.60 (1.2)	3.59 (1.40)	−0.01	3.17 (1.61)	2.79 (1.46)	−0.71		

Notes: Values are expressed as the median [IQR] or mean (SD); * *p* < 0.05 between-group analysis (Mann–Whitney U test); (°/s): degrees per second.

## Data Availability

The data presented in this study are available upon request from the corresponding author.
